# Simvastatin Impairs Growth Hormone-Activated Signal Transducer and Activator of Transcription (STAT) Signaling Pathway in UMR-106 Osteosarcoma Cells

**DOI:** 10.1371/journal.pone.0087769

**Published:** 2014-01-29

**Authors:** María Claudia Sandoval-Usme, Adriana Umaña-Pérez, Borja Guerra, Orlando Hernández-Perera, José Manuel García-Castellano, Leandro Fernández-Pérez, Myriam Sánchez-Gómez

**Affiliations:** 1 Hormone Laboratory, Department of Chemistry, Universidad Nacional de Colombia, Bogotá, Colombia; 2 Department of Clinical Sciences, Molecular and Translational Endocrinology Group, University of Las Palmas de Gran Canaria – Cancer Research Institute of The Canary Islands (ICIC), Las Palmas de Gran Canaria, Spain; 3 Associated Biomedical Unit of ULPGC-IIBM “Alberto Sols” - CSIC, Las Palmas de Gran Canaria, Spain; 4 Laboratory of Molecular Oncology, Research Unit, Complejo Hospitalario Universitario Insular Materno Infantil, CHUIMI, Las Palmas de Gran Canaria, Spain; China Medical University, Taiwan

## Abstract

Recent studies have demonstrated that statins reduce cell viability and induce apoptosis in various types of cancer cells. The molecular mechanisms underlying these effects are poorly understood. The JAK/STAT pathway plays an important role in the regulation of proliferation and apoptosis in many tissues, and its deregulation is believed to be involved in tumorigenesis and cancer. The physiological activation of STAT proteins by GH is rapid but transient in nature and its inactivation is regulated mainly by the expression of SOCS proteins. UMR-106 osteosarcoma cells express a GH-responsive JAK2/STAT5 signaling pathway, providing an experimental model to study the influence of statins on this system. In this study we investigated the actions of simvastatin on cell proliferation, migration, and invasion on UMR-106 cells and examined whether alterations in GH-stimulated JAK/STAT/SOCS signaling may be observed. Results showed that treatment of osteosarcoma cells with simvastatin at 3 to 10 µM doses decreases cell proliferation, migration, and invasion in a time- and dose-dependent manner. At the molecular level, although the mechanisms used by simvastatin are not entirely clear, the effect of the statin on the reduction of JAK2 and STAT5 phosphorylation levels may partially explain the decrease in the GH-stimulated STAT5 transcriptional activity. This effect correlated with a time- and dose-dependent increase of SOCS-3 expression levels in cells treated with simvastatin, a regulatory role that has not been previously described. Furthermore, the finding that simvastatin is capable of inducing SOCS-3 and CIS genes expression shows the potential of the JAK/STAT pathway as a therapeutic target, reinforcing the efficacy of simvastatin as chemotherapeutic drug for the treatment of osteosarcoma.

## Introduction

Statins inhibit 3-hydroxy-3-methylglutaryl CoA (HMG-CoA) reductase, the rate-limiting enzyme in the mevalonate biosynthetic pathway, and source of intermediates involved in protein farnesylation and geranylation [1]. These posttranslational modifications are vital for proper functioning of proteins Ras, Rho, Rac, and other small GTPases, which are involved in the regulation of several biological processes including cell proliferation, migration, viability, cell cycle, and invasiveness [2], making them important targets for understanding statin effects. Statins have been traditionally used to treat hypercholesterolemia and other cardiovascular diseases; however, recent studies have found that statins are able to induce apoptosis, thereby decreasing cell viability and proliferation of several cancer cell lines, including colorectal [3], prostate [4], pancreatic [5], breast cancer [6], and melanoma cells [7]. Treatment with atorvastatin sensitizes osteosarcoma cells to chemotherapy hence reducing cell survival [8]. In spite of the growing evidence of the effects of statins in different cell types, their molecular mechanisms are still unclear. Although RhoA and Ras family proteins have been the most investigated targets in statin research, several studies have linked statins to the Janus Kinases/Signal Transducers and Activators of Transcription (JAK/STAT) signaling pathway. This signaling pathway is an important regulator of cell proliferation, differentiation, survival, motility, and apoptosis [9]. Deregulation of this pathway has been found to directly contribute to oncogenesis and malignant transformation of several types of cancer [10]–[12]. JAK/STAT signaling is activated by a variety of hormones, cytokines and growth factors and it induces its own inactivation, mainly by Suppressors Of Cytokine Signaling (SOCS) protein family, acting in a negative feedback loop [13]. Their N-terminal and SH2 domains are responsible for competitive inhibition of signaling proteins by interaction with the JAKs or the receptors themselves [14]. Statins, including simvastatin, inhibited the JAK/STAT signaling pathway in cardiomyocytes [15] and vascular endothelial cells [16] besides upregulating mRNA and protein expression of SOCS-3 and SOCS-7 in the macrophage cell line RAW264.7 [17], [18].

Growth Hormone (GH) is a pleiotropic hormone that stimulates growth, mitogenesis, and proliferation in various tissues and cell types. It mainly activates JAK2 and both isoforms of STAT5, A and B. Several studies have elucidated the proliferative effects of GH on osteoblasts [19] as well as the anabolic effects on bone [20] and has been reported that long exposure to GH could act as predisposing factor in the development of metastatic osteosarcoma [21]. Recent studies report that humans with GH-receptor deficiency are protected from developing cancer, decreasing the susceptibility of cell to DNA damage and abnormal proliferation [22].

UMR-106 is a rat osteosarcoma cell line with osteoblast-like properties. It expresses a JAK2/STAT5 signaling system activated by GH [23], making it a suitable model to study GH signaling in osteoblasts. In a previous study, we examined the effects of simvastatin, a lipophilic statin, on UMR-106 and HTR-8/SVneo trophoblast cell lines. We found that simvastatin was able to decrease cell viability and induce apoptosis on both cell types [24]. We also found that simvastatin had an inhibitory action on RhoA and RhoB isoprenylation. RhoA, was observed to regulate STAT1 transcriptional activity, and simvastatin treatment was associated with decreased STAT1 activation and transcriptional activity [25]. The aim of the present study was to further investigate the molecular mechanism modulated by statins on cancer cells, by examining the effects of simvastatin on the JAK/STAT/SOCS signaling pathway activated by GH in UMR-106 cells, specifically whether simvastatin was able to decrease GH signaling, modulating the JAK/STAT pathway in its activation, transcriptional activity and regulation by SOCS proteins.

## Materials and Methods

### Cell culture and treatments

UMR-106 rat osteosarcoma (ATCC CRL-1661), BRL-4 (buffalo rat liver cells) [26] and MCF-7 (human breast cancer) (ATCC HTB-22) [27] were grown in DMEM. All media were supplemented with 10% Fetal Bovine Serum (FBS), 2 mM glutamine, 100 units/ml penicillin, and 100 µg/mL streptomycin. Cells were maintained at 37°C in a humidified atmosphere with 5% carbon dioxide. For simvastatin (Sigma, USA) treatment, cell media was refreshed and simvastatin or ethanol (final concentration 0.01%) was added in the indicated concentration. Bovine Growth Hormone (GH, AFP-10325C, National Hormone and Peptide Program, NHPP, USA) was employed in all assays

### Cell proliferation assays

In order to measure proliferation rate, Bromodeoxyuridine (BrdU) incorporation assay was carried out using a cell proliferation ELISA BrdU kit (Roche Diagnostics, USA) according to manufacturer's instructions. Briefly, UMR-106 cells were seeded at 2500 cells/well in 96-well plates in FBS-supplemented media. After 24 h of incubation, cells were labeled with 10 µM BrdU and incubated for an additional 4 h at 37°C. Labeling medium was removed from the microplates, cells were dried and fixed, and cellular DNA was denatured with FixDenat solution (Roche Diagnostics, USA) for 30 min at room temperature. A mouse peroxidase-conjugated anti-BrdU monoclonal antibody was added to each well and plates were incubated again at room temperature for 2 h. After plates were washed, they were incubated with substrate solution containing hydrogen peroxide, luminol and 4-iodophenol. The immunocomplex was quantified by luminescence emission in a luminometer Fluoroskan Ascent FL (Labsystems). The assay was performed in triplicates and results were expressed as cell proliferation percentage, taking control cells as 100% proliferation ± SEM.

### Real Time–Cell electronic sensing (RT-CES)

Electrical impedance was detected in a real-time electronic sensing system RT-CES (xCELLigence, Roche, USA) to assay the dynamic cell response to different doses of simvastatin. The cell growth measured by this system is comparable to the actual cell number and is expressed as cell index [28]. UMR-106 cells (5000 cells/well) were seeded in FBS-supplemented medium. After 18 h, different doses of simvastatin (0.1–10 µM) were added and measurements were done in three replicates every five minutes for a total period of 72 h.

### Cell viability assay

Cell viability was assayed by measuring the mitochondrial reduction of the tetrazolium salt 3-(4,5-methylthiazol-2-yl)-2,5-diphenyl-tetrazolium bromide] (MTT) [29]. MCF-7 and BRL4 cells were seeded in 96-well plates in FBS supplemented medium at a cell density of 2×10^4^ cells/well. Twenty-four hours later, simvastatin was added to the medium at the indicated concentrations and cells were cultured for the indicated times. MTT (0.5 mg/ml) was added to each well for the last four hours and incubated at 37°C in the dark. The medium was then discarded and the formazan precipitate was solubilized by addition of 20% SDS in 0.02N HCl for 12–16 h. The optical density was measured at 595 nm with the iMark Microplate Reader (BioRad, USA). Data was expressed as percent growth above the level in controls and the results in figures were plotted as mean ± SEM of each test point from 3 replicates. IC_50_ was determined from a plot when fitted to a sigmoidal dose-response curve by using Graph Pad Prism version 5.0 for Windows, (Graph Pad Software, La Jolla California USA, www.graphpad.com).

### Wound healing assay

UMR-106 cells were seeded on 12-well plates and grown to a confluent monolayer. Cell monolayer was scrapped with a micropipette tip, washed with PBS and incubated for 48 h at 37°C in 10% FBS-supplemented DMEM, with or without simvastatin. Cells were photographed 48 h after the wound was made. Before imaging, cells were washed with PBS and media were refreshed. Images were acquired using a Nikon camera fitted to an inverted microscope.

### Cell migration and invasion assays

Cell migration and invasion was evaluated in uncoated or matrigel-coated Boyden chambers, respectively, following manufacturer's instructions (DB Biosciences, Bedford, MA, USA). Lower chambers were filled with FBS supplemented medium and final concentration of simvastatin. Cell invasiveness was measured in a similar manner with slight modifications in the protocol. Matrigel transwell chambers were hydrated with 500 µL DMEM for 4 h prior to use in the upper and lower compartment. Before seeding cells, medium was removed and the lower compartment was filled with 600 µL of medium as indicated. Immediately afterwards, in both cases, 2500 cells were added to the upper chamber, where they remained for 36 hours. Non-migrating cells were swabbed from the upper chamber and its lower face was fixed with paraformaldehyde 4%, and dyed with a 0.5% crystal violet methanol solution. The 8 µm-pore polycarbonate membranes were cut off from transwells and crystals were dissolved in 10% acetic acid. Absorbance was measured at 540 nm in duplicate.

### Preparation of whole cell extract and Western blot analysis

UMR-106 cells were grown to 80% confluence in complete medium and then, they were serum-starved over night before the experiments. Treatments, at times and concentrations indicated, with simvastatin and GH were performed in serum-free medium. After treatments, cells were rinsed in ice-cold PBS and scraped with RIPA lysis buffer (25 mM Tris-HCl pH 7.6, 150 mM NaCl, 1% NP-40, 1% sodium deoxycholate, 0.1% SDS; Thermo Scientific, Rockford, IL, USA) supplemented with 1X Halt protease and 1X phosphatase inhibitor cocktails (Thermo Scientific, Rockford, IL, USA), and incubated on ice for 30 min. The supernatant obtained after centrifugation was used as whole-cell extract. An aliquot of each extract was preserved for protein quantification by bicinchoninic acid assay (Thermo Scientific, Rockford, IL, USA). Proteins were solubilized in sample buffer containing 62.5 mM Tris-HCl, pH 6.8, 2.3% (wt/vol) SDS, 10% (vol/vol) glycerol, 5% (vol/vol) β-mercaptoethanol, and 0.001% (wt/vol) bromophenol blue and boiled at 95°C for 5 min. Equal amounts (50 µg) of each sample were electrophoresed on 8–10% sodium dodecyl sulfate – polyacrylamide gel electrophoresis (SDS-PAGE) and transferred to nitrocellulose membranes (Invitrogen, Barcelona, Spain). Ponceau S staining solution (0.5% Ponceau S and 1% glacial acetic acid in water) was used after transfer to verify equal protein loading in the control and treated samples (data not shown). Membranes were blocked with 5% blotting grade blocker nonfat dry milk in Tris Buffered Saline with 0.05% Tween 20 (TBST; blotto-blocking buffer) for at least 1 h at room temperature. Then, they were washed twice in TBS with 0.1% Tween 20 (TBST-0.1). Membranes were then incubated over night at 4°C with the appropriate anti-phospho-antibodies (monoclonal rabbit anti-phospho-Tyr^1007/1008^-Jak2 antibody, polyclonal rabbit-anti-phospho-Tyr^694^-Stat5 antibody, polyclonal rabbit-anti-phospho-Ser^726/731^-Stat5 antibody, polyclonal rabbit anti-phospho-Tyr^705^-Stat3 antibody and polyclonal rabbit-anti-phospho-Tyr^701^-Stat1 antibody (all from Cell Signaling Technology, Danvers, MA) all diluted (1∶1000) in 1% bovine serum albumin (BSA)-1% blotto in TBST. Antibody-specific labeling was revealed by incubation with an HRP-conjugated goat anti-mouse secondary antibody (1∶5000) (sc-2031, Santa Cruz Biotechnology, Santa Cruz, CA, USA) or an HRP-conjugated goat anti-rabbit secondary antibody (1∶5000) (sc-2030, Santa Cruz Biotechnology, Santa Cruz, CA, USA) and visualized with the Immu-Star™ WesternC™ kit (Bio-Rad Laboratories, Hercules, CA, USA). Membranes were stripped of bound antibodies by incubation in stripping buffer (Thermo Scientific, Rockford, IL, USA) at room temperature for 30 min with agitation. Membranes were washed for 3×10 min in TBST-0.1, blocked with blotto-blocking buffer for at least 1 h and re-probed with the corresponding anti-total kinase antibodies (monoclonal rabbit anti-Jak2 antibody, monoclonal mouse anti-Stat3 antibody, polyclonal rabbit anti-Stat1 antibody (all from Cell Signaling Technology, Danvers, MA) and polyclonal rabbit anti-Stat5 antibody (Santa Cruz Biotechnology, Santa Cruz, CA, USA)) all diluted (1∶1000) in blotto-blocking buffer. To check for differences in loading and transfer efficiency across membranes, an antibody directed against β-actin (monoclonal mouse anti-β-actin antibody, sc-81178, Santa Cruz Biotechnology, Santa Cruz, CA, USA) was used to hybridize with all membranes previously incubated with the respective phospho and total kinase antibody. Specific bands were visualized using the ChemiDoc XRS System (Bio-Rad Laboratories, Hercules, CA, USA) and analyzed with the image analysis program Quantity One (Bio-Rad Laboratories, Hercules, CA, USA).

### Cell transfection assays

STAT5, STAT3 and STAT-1/-3 regulated reporter plasmids pSPI-GLE1-Luc [26], pSTAT3, pISRE, respectively, (donated by Dr. Juan Carlos Lacal, Instituto de Investigaciones Biomédicas “Albero Sols”, CSIC, Madrid, Spain), SOCS-1, SOCS-2 and SOCS-3 (pSOCS-1, pSOCS-2 y pSOCS-3, donated by Dr. Amilcar Flores Morales (CMM, KI, Sweden) were used to examine transcriptional activity in UMR-106 cells. Cells were grown to 80% confluence in 6-well plates and were serum-deprived before transfection. Then, 1 µg of plasmid DNA was transfected overnight using Metafectene® (Biontex), according to manufacturer's instructions. After transfection, cells were incubated for 8 h before treatment with serum-free medium with 10 µM simvastatin or vehicle for 16 h. Then, medium was changed to 50 nM GH or FBS-supplemented medium for additional 16 h. Cells were extracted with Passive Lysis Buffer solution (Promega) and luciferase activity was determined by using the Luciferase Assay System (Promega). Luciferase activities were measured in duplicate in microplate reader Fluoroskan Ascent FL (Labsystems). Cells transfected with an empty plasmid were used as control in the assay. Results were obtained as relative luciferase units (RLU) per mg of protein and expressed as fold induction to control cells.

Cells were co-transfected, where indicated, with 0.05 µg of STAT5 S730A plasmid [30] -which expresses phosphorylated STAT5 protein, once is first activated, and 1 µg of SPI plasmid. After overnight transfection, procedure followed as in the other transfections.

### Gene expression analysis by Real Time quantitative-PCR (qPCR)

Total RNA was isolated from UMR-106 cells using Trizol (Invitrogen, USA) according to manufacturer's protocol. RNA yields were measured by UV absorbance, and the quality of total RNA was analyzed by agarose electrophoresis. mRNA expression levels of genes were measured using qPCR. Briefly, 2 µg of total RNA were treated with RNase-free DNase I (Promega) to remove genomic DNA and were reverse-transcribed using iScriptTM reverse transcriptase kit (Bio-Rad Laboratories). Two microliters of cDNA served as a template in a 20-µL qPCR reaction mix containing the primers and SYBR Green PCR Master Mix (Diagenode, Belgium). Quantification of gene expression was performed with an ABI PRISM® 7000 SD RT-PCR (Applied Biosystems) according to the manufacturer's protocol. Data were extracted and amplification plots generated with ABI SDS software. The level of individual mRNA measured by qPCR was normalized to the level of the reference gene cyclophiline by using Pfaffl method [31]. For graphing purposes, the relative expression levels were scaled such that the expression of the vehicle-matched control groups equaled one. PCR primers for SOCS-2 (forward 5′-GACGGGAAATTCAGATTGGA-3′; reverse 5′-AATGCTGAGTCGGCAGAAGT-3′) SOCS-3 (forward 5′-CCTTTGAGGTTCAGGAGCAG-3′; reverse 5′-CGTTGACAGTCTTCCGACAA-3′), CIS (forward 5′-CCACCCCAGCTACCTGTTTA-3′, reverse 5′-CGTACAGGAGGCCACGTAAT-3′), and cyclophiline (forward 5′-GGTGACTTCACACGCCATAA-3′; reverse 5′-AGCCACTCAGTCTTGGCAGT-3′) were obtained from Thermo Scientific. Gene expression analyses were performed in cells grown in serum free media containing variable doses of simvastatin (2, 10, 20 µM) for 8 h prior stimulation with 50 nM GH, for 1 h. Cells treated with vehicle (0.01% ethanol) were used as control.

### Statistical analysis

The significance of differences between the groups was tested by either a two-tailed Student's *t* test or a one-way ANOVA, which was followed by post hoc comparisons of the group means according to the Graph Pad Prism 5 program (Graph Pad Software, San Diego, CA). Statistical significance was reported if *p*<0.05 was achieved.

## Results

### Simvastatin decreases the proliferation of osteosarcoma cells

**Figure 1:**
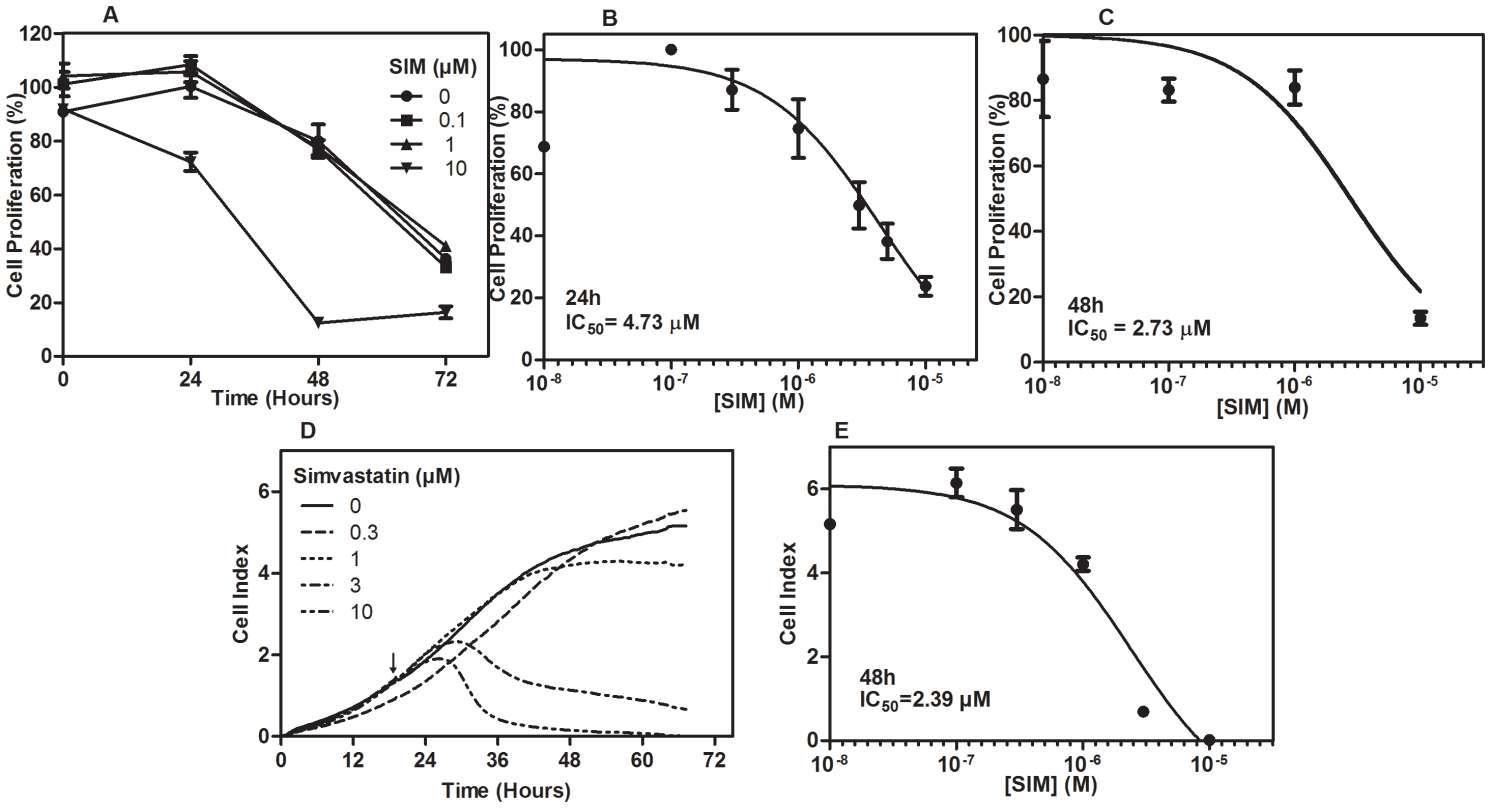
Effects of simvastatin treatment on proliferation and cytotoxicity of UMR-106 osteosarcoma cells. Cells were treated with increasing doses of simvastatin (SIM) as indicated and cell proliferation was measured using a bromodeoxyuridine (BrdU) incorporation assay. Proliferation levels were measured at 24, 48 and 72 hours and results were expressed as percentage to control cells (100%) (A). Statistical differences were observed after 24 h at 10 µM (p<0.01). IC_50_ values obtained at 24 (B) and 48 hours (C) after simvastatin treatment. Values are the mean ± SEM. Cell index was measured by a dynamic monitoring response (RT-CES assay) to increasing simvastatin doses for the indicated time period. The arrow indicates the time when simvastatin was added (D). IC_50_ value was calculated at 48 hours (E).

We studied the effects of simvastatin on proliferation of osteosarcoma cells, by measuring the amount of BrdU incorporated into DNA (Fig. 1). Figure 1A shows that simvastatin caused a time- and dose-dependent inhibition on UMR cells proliferation. We found a significant reduction in cell proliferation after 24 h treatment with 10 µM simvastatin (p<0.01). IC_50_ of simvastatin was 4.73 µM at 24 h (Fig. 1B) and 2.73 µM at 48 h (Fig. 1C). Moreover, we applied an electrical impedance detection method (RT-CES) to study the dynamic response to increasing simvastatin. Simvastatin reduces cell index, which is a marker of changes in cell adhesion and integrity [28], confirming the anti-proliferative effects of simvastatin on UMR-106. In absence of simvastatin, cell index increased over the experimental period of 72 h (Fig. 1D). Effects of simvastatin were noticeable after 7 h of treatment, as cell index started to drop at doses higher than 1 µM. It is likely that cells treated with simvastatin at concentrations of 1 µM or lower, might recover from the inhibitory effects of the drug. On the contrary, at higher doses of statin, cells are detached from the surface which might suggest changes in cell adhesion mechanisms, however further studies are required to confirm that. These assays were performed in presence of FBS; therefore we exclude a possible protective effect of FBS that might neutralize the actions of simvastatin over cells. After 48 hours, the IC_50_ value was calculated to be 2.39 µM (Fig. 1E).

**Figure 2:**
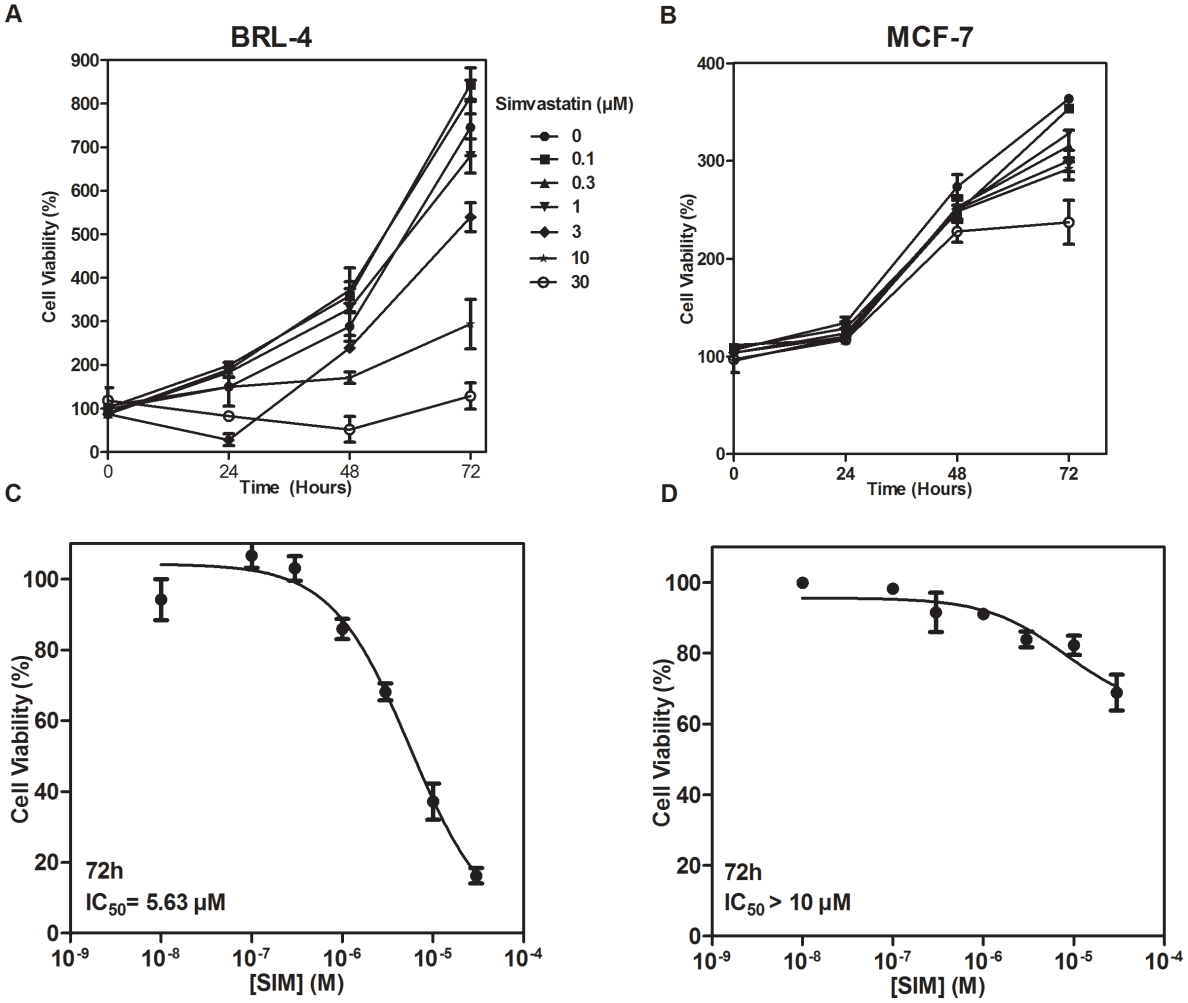
Effects of simvastatin on cell viability in BRL-4 and MCF-7 cells. BRL-4 cells (A) and MCF-7 cells (B) were treated with increasing doses of simvastatin for 24, 48 and 72 hours as indicated. Cell viability was measured using the MTT method and expressed as percentage to control cells (100%). IC_50_ values were calculated after 72 hours of simvastatin treatment for BRL-4 (C) and MCF-7 (D) cells. Values are the mean of three replicates ±SEM.

We previously reported [24] the effects of simvastatin on cell viability of UMR-106 osteosarcoma cells (IC_50_ 2.7 µM at 48 h), which aimed us to investigate whether the same behavior was seen in other cell types. We used a non-cancerous BRL-4 cell line stably transfected with the rat GH receptor complementary DNA (cDNA), previously shown to respond to GH [26] and MCF-7 breast cancer cells which display a high proliferative but less invasive phenotype compared to UMR-106 cell line, and simvastatin has been shown to induce cell cycle arrest and apoptosis [6]. Dose-response cell viability analysis for BRL-4 and MCF-7 cell lines showed a decrease in cell viability with increasing doses (0.1 to 30 µM) of simvastatin (Fig. 2A, B). However, both cell lines were less affected by the drug in comparison to osteosarcoma cells, as higher doses of simvastatin were needed to reduce the growth of BRL-4 and MCF-7 cells after 72 h of exposure to the statin (Fig. 2C, D).

### Simvastatin decreases migration and invasion of osteosarcoma cells

**Figure 3:**
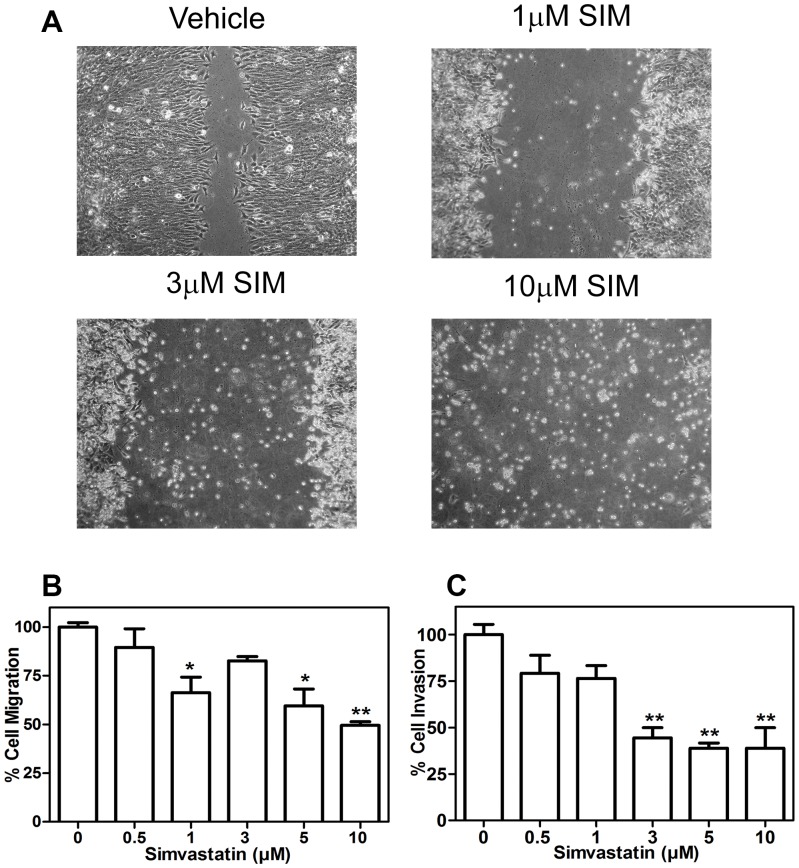
Migration and invasion levels of Simvastatin UMR-106 treated cells. UMR-106 monolayer were scraped with a pipette tip and incubated in the absence or presence of simvastatin. Wounds were photographed at 48 h after treatment and a representative result of three experiments is shown to schematize the inhibitory effect of simvastatin on migration rate (A). Cells were seeded in the upper level of 8 µm-Boyden chamber uncoated (B) or coated with matrigel (C), attracted by simvastatin and 10% FBS supplemented medium in the lower chamber. After 48 h migrating cells were stained with crystal violet methanol solution, the 8 µm-pore membranes were cut off and crystals were dissolved in 10% acetic acid. Absorbance solution was measured at 540 nm. Quantitative response was expressed as percentage of the control ± SEM of two replicates. *a* means p<0.001, *b* means p<0.05 vs control, after a one-way ANOVA analysis.

Functional implications of simvastatin on osteosarcoma cell migration were examined by two different methods. Firstly, for the wound healing assay, cells were scrapped across a confluent monolayer of cells (Fig. 3A). Results showed that UMR-106 cells treated with vehicle were capable of migrating towards the gap, while cells treated with increasing doses of simvastatin, were unable to migrate at the same rate (Fig. 3A). Cells exposed to doses of simvastatin above 1 µM, detached from the surface and therefore, the wound did not heal. Similar results were observed when cell motility was evaluated by the Boyden chamber assay (Fig. 3B). Furthermore, invasive capacity of osteosarcoma cells was also affected by the statin, as is shown in the assays performed in Matrigel-coated Boyden chambers (Fig. 3C). Simvastatin significantly reduced cell invasiveness at doses of 3 µM, compared to vehicle-treated control cells.

### Simvastatin impairs GH-induced JAK2/STAT5 phosphorylation

**Figure 4:**
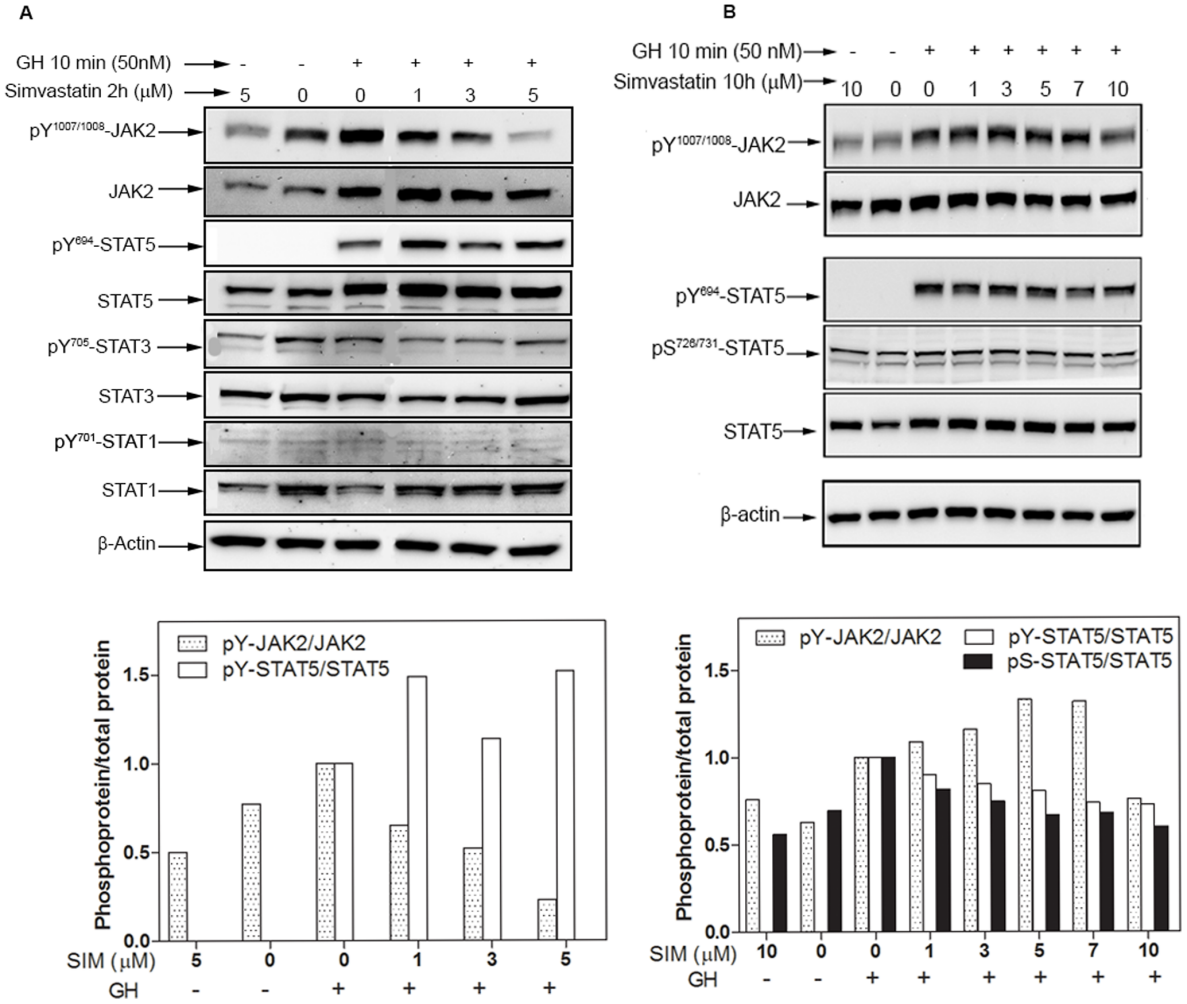
Effect of simvastatin on JAK/STAT phosphorylation in UMR-106 cells. Cells were pretreated with simvastatin as indicated, and then stimulated with 50(A) or 10 h (B) of simvastatin incubation. β-actin levels were used as a load control protein.

To elucidate the molecular mechanism of simvastatin on cancer cells, we focused our study on the GH-activated JAK/STAT signaling pathway. Firstly, we found that after 10 min of 50 nM GH, JAK2 phosphorylation (pY^1007/1008^-JAK2) was attenuated in cells pretreated for 2 h with increasing doses of simvastatin up to 5 µM (Fig. 4A). Unexpectedly, STAT5 tyrosine phosphorylation response to increasing doses of simvastatin showed an unclear trend, and STAT1 and STAT3 phosphorylation levels were not affected by simvastatin treatment at this time (Fig. 4A). Therefore, we explored the effects of simvastatin at longer time of exposure and at higher doses. Our findings show that GH-induced JAK2 phosphorylation was reversed only by a long incubation period with 10 µM simvastatin, and that at lower concentrations the regulation was different, suggesting that after long periods, other processes can occur, inducing other mechanisms that affect JAK2 phosphorylation pattern (Fig. 4B).

Moreover, when cells were grown in presence of increasing doses of the statin (1 to 10 µM) for 10 h prior to GH stimulation, we observed a moderate dose-dependent attenuation in STAT5 tyrosine phosphorylation (Fig. 4B). In addition, STAT5 activation has been shown to be modulated by serine phosphorylation [32]. To address this aspect, we examined the effect of simvastatin on pS^726/731^-STAT5 and interestingly a reduction in the phosphorylation level was already detected with 1 µM simvastatin, which further decreased with higher doses of the statin. After 10 µM treatment, pS-STAT5 reaches its basal value, suggesting that simvastatin annuls the effect of GH (Fig. 4B).

### STAT transcriptional activity is modulated by simvastatin

**Figure 5:**
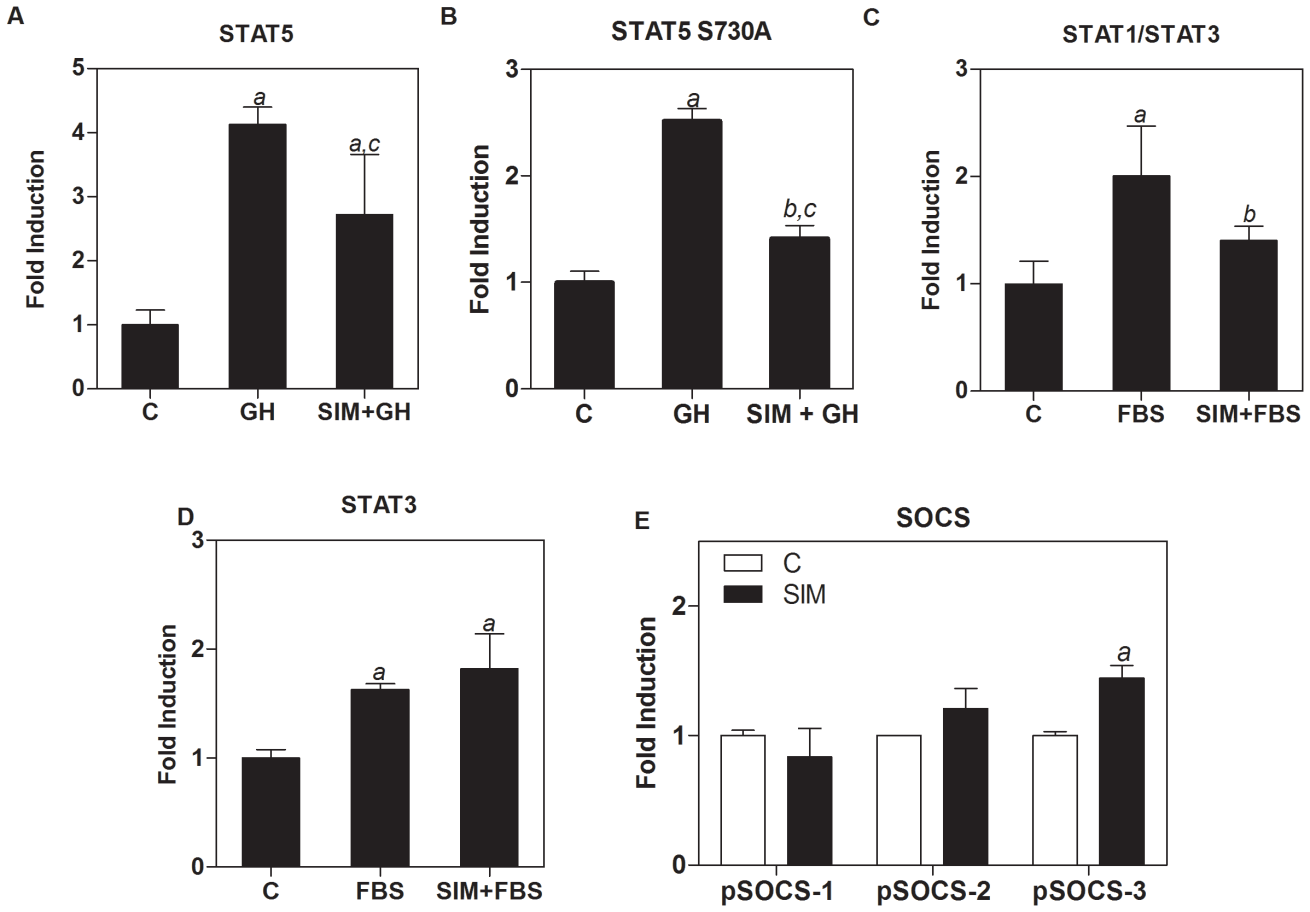
Effect of simvastatin-pretreated UMR-106 cells on GH- or FBS-induced transcriptional activity. After transfection, cells were incubated for 8-free medium with 10 µM simvastatin or vehicle for 16 h. Then, cells were stimulated with 50 nM GH or 10%FBS –as indicated- for 16 hours. Cells were transiently transfected with pSPI-GLE-Luc reporter plasmid activated by STAT5 (A) and co-transfected with pSTAT5 S730A (B); pISRE-Luc reporter plasmid activated by STAT1/3 (C), pSTAT3-Luc reporter plasmid activated by STAT3 (D) or pSOCS-1, -2, -3 gene promoter plasmids (E). Treatments are as follows: C  =  Non-stimulated transfected control cells; SIM  =  Simvastatin. Luciferase expression results are expressed relative to non-treated cells. *a* means p<0.001, vs control cells; *b* p<0.05, vs control cells; *c* p<0.05 vs GH-treated cells. Values are mean ± SEM, after a one-way ANOVA analysis.

Next we investigated whether simvastatin had an effect on GH-induced STAT5-dependent transcriptional activity. We transiently transfected UMR-106 cells with a SPI-GLE-Luc reporter plasmid activated by STAT5. Figure 5A shows a 4-fold induction of STAT5 transcriptional activity by GH compared to non-stimulated control cells. However, cells treated with 10 µM simvastatin showed a 40% inhibition of GH-dependent STAT5 transcriptional activity. Consistent with these findings, we found that cells co-transfected with both the SPI-GLE-Luc reporter plasmid and the pSTAT5 S730A plasmid, showed a 50% reduction in transcriptional activity after simvastatin treatment (Fig. 5B).

Moreover, we evaluated STAT1 and STAT3 transcriptional activity by using a pISRE-Luc reporter plasmid. We found that 10 µM simvastatin significantly decreased transcriptional activity induced by FBS (Fig. 5C) and that a lower dose of 1 µM did not cause a significant change in transcriptional activity (data not shown). We further analyzed the effects of simvastatin on FBS-induced STAT3 transcriptional activity using a STAT3-Luc reporter plasmid. Results indicate that simvastatin does not induce a significant change in FBS-induced activity (Fig. 5D). Taking into account that SOCS proteins negatively regulate the JAK/STAT pathway, we analyzed the effects of simvastatin on the SOCS gene promoters. We found that simvastatin modulates SOCS-3 promoter, increasing the activity of the reporter plasmid (Fig. 5E). In contrast, SOCS-1 or SOCS-2 promoters did not show a significant activation by the statin in comparison with the empty plasmids.

### Simvastatin induces SOCS-3 and CIS expression

**Figure 6:**
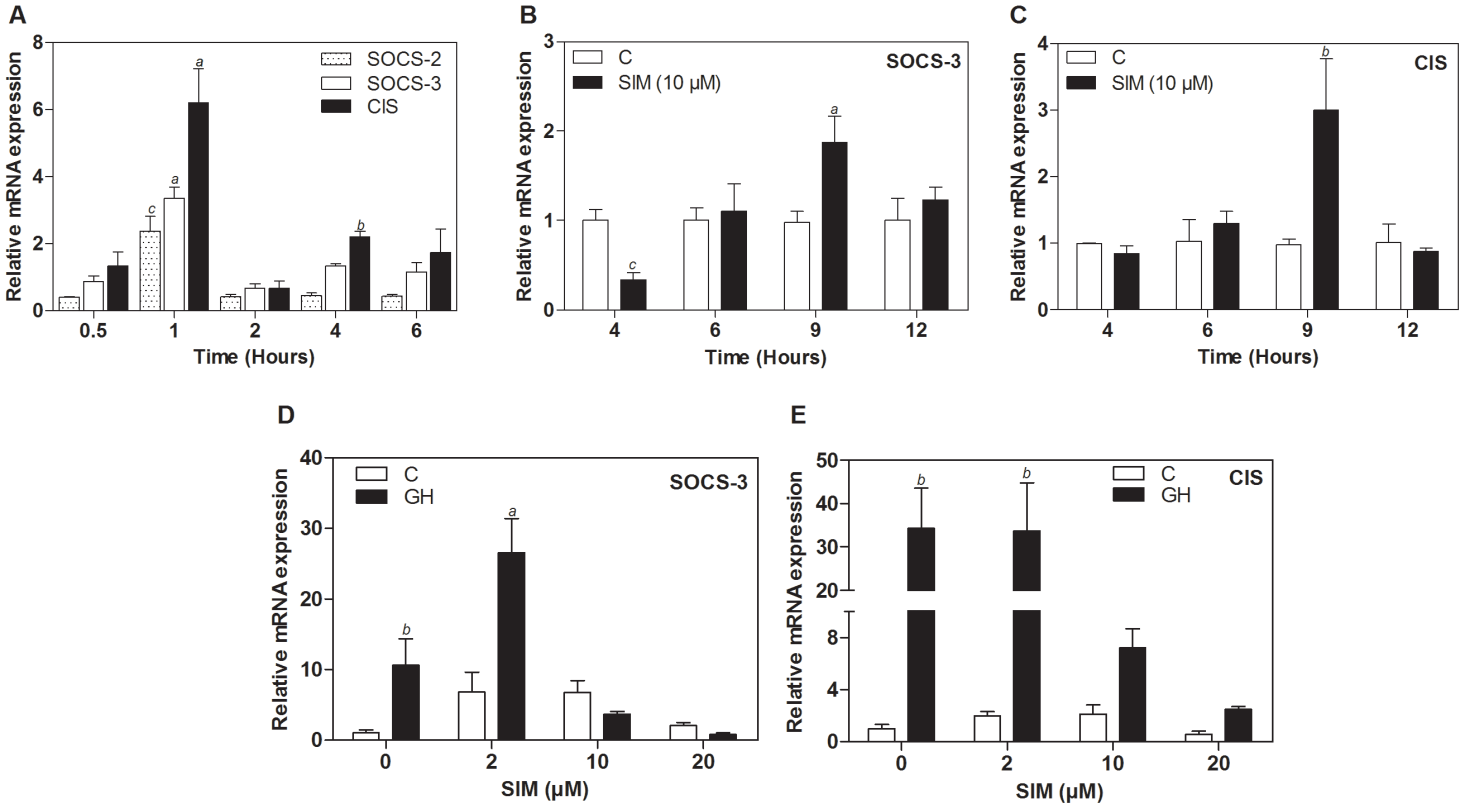
Effect of simvastatin and GH treatment of UMR-106 cells on SOCS gene expression. (A) mRNA expression after GH treatment in the time points indicated for SOCS-2, SOCS-3 and CIS. Gene expression of SOCS-3 (B) and CIS (C) after 10 µM simvastatin treatment in the time points indicated. Gene expression of SOCS-3 (D) and CIS (E) in cells treated with simvastatin for 8 hours in increasing doses prior to stimulation with GH 50 nM for 60 minutes. Treatments are as follows: C  =  Non-stimulated control cells; SIM  =  Simvastatin. Values are the mean ± SD (n = 3) and normalized to the level of the reference gene cyclophiline. *a* p<0.05, *b* p<0.005, *c* p<0.001, after a two-way ANOVA analysis.

As our results showed an activation of SOCS-3 promoter, we investigated whether simvastatin had any regulatory role at the transcriptional level of SOCS genes, evaluating UMR-106 cells response to GH stimulation and simvastatin treatment. First, we found that GH induced mRNA expression of CIS, SOCS-2 and SOCS-3 with maximum levels at 1 h of hormone treatment (Fig. 6A). Consistent with the results mentioned previously, we found that treatment with simvastatin induced mRNA levels of SOCS-3 and CIS genes in a time-dependent manner (Fig. 6B and C). Moreover, we also found a dose-dependent effect of simvastatin. When 2 µM simvastatin treatment for 8 h was combined with 50 nM GH, SOCS-3 mRNA expression was enhanced (Fig. 6D). Western blot analysis also showed a higher SOCS-3 protein basal level in cells after 10 h exposure to simvastatin (Result not shown). CIS transcription was not further increased, more likely due to the already high levels of expression of this gene, compared to other SOCS genes (Fig. 6E). For both genes, we observed higher concentrations of simvastatin (10 to 20 µM) may affect cell viability and therefore mRNA levels were not as elevated as with lower statin doses (Fig. 6D and E)

## Discussion

Statins are widely used in the treatment of hypercholesterolemia and other cardiovascular diseases. These drugs inhibit the mevalonate pathway, by targeting the HMG-CoA reductase, the rate-limiting step enzyme in cholesterol synthesis [33]. Recent studies have demonstrated that statins, besides their cholesterol-lowering effects, reduce cell proliferation and viability and induce apoptosis in various types of cancer cells [3]–[6]. In this study we found that simvastatin, a lipophilic statin, decreases cell proliferation, migration and invasion levels in UMR-106 rat osteosarcoma cells. This is consistent with previous results in our group, where we found that cell viability is decreased in a dose-dependent manner [24].

Interestingly, when comparing to a non-cancerous rat hepatocyte BRL-4 cell line, results showed the higher sensitivity of osteosarcoma cells, suggesting the potential anti-tumor actions of the statin. In addition, we examined the antiproliferative effects of simvastatin on MCF-7 human breast cancer cells, finding for this type of cells a lower sensitivity towards the statin. Our results are consistent with previous studies [6], [34], where MCF-7 cells were found to be more resistant to statin actions than other breast cancer and head and neck cancer cell lines. MCF-7 cells are poorly invasive, as compared to UMR-106 cells, which suggests that statins might target more invasive cell types and therefore, have a great potential as anticancer agents. In contrast to hydrophilic statins, hydrophobic simvastatin enters cells by free diffusion and is expected to affect a wide variety of organs and tumor cells. Although the simvastatin concentration required to reduce osteosarcoma cell growth and invasion *in vitro* may not be therapeutically achieved *in vivo*, daily intake of the statin for long periods of time could improve the antitumor efficacy of the drug.

Here we found three cell lines with different responses to simvastatin treatment, suggesting that differences in their signaling might account for their different behavior in response to simvastatin. It is necessary to further investigate whether mutations or other protein modifications might be important for statin response, which could target specific types of cancer cells. This also applies for the marked reduction in cell adhesion, migration and invasion observed in the presence of the statin in osteosarcoma. In the case of wound healing, cells treated with high simvastatin doses, not only were unable to migrate at all, but were clumping above the surface; this is in line with our findings in RT-CES, which very likely means that cell adhesion mechanisms are impaired, probably by alterations on membrane receptors and transmembrane proteins such as integrins [35]. Consequently, we cannot disregard the pro-apoptotic effects that simvastatin in doses higher than 3 µM might exert on cells.

In previous studies, these biological effects have been linked to the Rho and Ras family proteins, due to their direct association with statins and their inhibition effects on farnesylation and geranylation [36]. However, several pleiotropic effects have not been entirely elucidated and cannot be explained by direct alterations in the Rho and Ras family members.

The effects of simvastatin on other signaling pathways are currently under extensive investigation, including the JAK/STAT pathway. Here, we demonstrate that simvastatin modulates JAK2 phosphorylation in response to the statin. Previously, Zhang et al reported that a decreased JAK2 phosphorylation might be associated with changes in lipid raft composition, which destabilize the receptor- tyrosine kinase complex [18]. It is plausible to consider that the inhibitory effects of simvastatin in cholesterol synthesis, besides lowering raft cholesterol content and isoprenoid derivatives production, could also interfere with the expression of membrane proteins that require post-translational modifications derived from the mevalonate pathway. Therefore, it seems probable that the reduced JAK2 phosphorylation observed on GH-stimulated cells, pretreated with the statin, may be due to changes in membrane lipid composition as a result of the inhibitory effect of the drug.

We found a reduction in GH-induced STAT5 serine phosphorylation, along with a decrease in GH-stimulated STAT5 transcriptional activity in the presence of simvastatin. It has been found that tyrosine and serine phosphorylation are required to reach the highest STAT5 transcriptional activity [32]. In contrast to the extensive research on tyrosine phosphorylation and its function in dimerization, nuclear translocation and transcriptional activity, the role of serine phosphorylation has not been entirely elucidated [37], [38]. Park *et al* found two serine phosphorylation sites in STAT5a that can be constitutively phosphorylated, Ser^725^ and Ser^779^; while STAT5b can only be phosphorylated at Ser^730^ by GH, Prolactin and other cytokines [37]. Moreover, in several lymphoid tumors cell lines, constitutive phosphorylation of a newly identified cytokine-inducible Ser^193^ site within human STAT5b was found, supporting the possible involvement of serine phosphorylation in cancer cells [32].

Our results demonstrate an approximately 40% decrease in GH-stimulated STAT5-luciferase reporter gene activity, in osteosarcoma cells treated with the statin, which could be explained by the decrease in pS^726/731^-STAT5 observed in presence of simvastatin. The mechanism by which statins might interfere with STAT5 activation and nuclear localization and whether they interact with some cytoplasmic proteins to drive their biological actions remains undetermined and opens a new field of investigation.

The JAK2/STAT5 pathway is regulated by different mechanisms, including a negative feedback loop through the induction of SOCS proteins [39]. GH has been shown to induce the expression of different combinations of SOCS-1 to -3 and CIS. While the primary SOCS-1 and SOCS-3 interaction has been described with critical phosphotyrosines located within the catalytic loop of the JAK2 molecule, SOCS-3 has been shown to interact with high affinity regions within the receptor subunits [40]. CIS and SOCS-2 also bind to receptor phosphotyrosines and inhibit signaling by competing with STAT molecules for recruitment to the receptor complex [41]. Among the prevailing mechanisms by which SOCS proteins inhibit cytokine signaling, their targeting of signaling molecules for proteosomal degradation has received great attention [42]. This raises the possibility that SOCS proteins may inhibit signaling by functioning as adaptors for an E3 ubiquitin ligase complex.

We show here the GH-induced expression of SOCS-2, SOCS-3 and CIS in UMR-106 cells with peak values after 1 hour of exposure to the hormone, in agreement with the study by Morales et al. [43]. Furthermore, our results show that simvastatin induces the SOCS-3 and CIS expression in UMR-106 cells, in a time and dose dependent manner. To our knowledge this is the first report where simvastatin has been found to induce SOCS-3 and CIS gene expression in cancer cells. These findings might explain the effects of simvastatin in the GH-induced JAK/STAT pathway.

Furthermore, we found that pretreatment with simvastatin, at doses compatible with cell viability (e.g. 2 µM) increase GH-induced SOCS-3 expression. Both SOCS-3 and CIS have been shown to inhibit or decrease GH activation of STAT5 and STAT5-dependent transcriptional activity [44]. Thus, it is possible that simvastatin-induced expression of SOCS-3 and CIS may contribute to the reduced STAT5 transcriptional activity we observed in UMR-106 cells. However, further studies knocking down SOCS genes could clarify the modulation of GH responses to simvastatin.

In conclusion, our results demonstrate that simvastatin has several biological activities on UMR-106 osteosarcoma cells, displaying osteosarcoma high sensitivity to the statin actions, in comparison with other cancer types. At the molecular level, although the mechanisms used by simvastatin are not entirely clear, the effect of the statin on the reduction of JAK2 and STAT5 phosphorylation levels, may partially explain the decrease in the GH-stimulated STAT5 transcriptional activity. This effect correlated with a time- and dose-dependent increase of SOCS-3 expression levels in cells treated with simvastatin, a regulatory role that has not been previously described. Furthermore, the finding that simvastatin is capable of inducing SOCS-3 and CIS gene expression, shows the potential of the JAK/STAT pathway as a therapeutic target, reinforcing the efficacy of simvastatin as chemotherapeutic drug for the treatment of osteosarcoma.
