# Correction: Simvastatin Impairs Growth Hormone-Activated Signal Transducer and Activator of Transcription (STAT) Signaling Pathway in UMR-106 Osteosarcoma Cells

**DOI:** 10.1371/journal.pone.0091619

**Published:** 2014-02-28

**Authors:** The PLOS ONE Staff

The name of the fourth author is incorrect. The correct name is: Octavio Hernández-Perera.
